# Structural color printing based on plasmonic metasurfaces of perfect light absorption

**DOI:** 10.1038/srep11045

**Published:** 2015-06-05

**Authors:** Fei Cheng, Jie Gao, Ting S. Luk, Xiaodong Yang

**Affiliations:** 1 Department of Mechanical and Aerospace Engineering, Missouri University of Science and Technology, Rolla, MO 65409, USA; 2 Center for Integrated Nanotechnologies, Sandia National Laboratories, Albuquerque, NM 87185, USA

## Abstract

Subwavelength structural color filtering and printing technologies employing plasmonic nanostructures have recently been recognized as an important and beneficial complement to the traditional colorant-based pigmentation. However, the color saturation, brightness and incident angle tolerance of structural color printing need to be improved to meet the application requirement. Here we demonstrate a structural color printing method based on plasmonic metasurfaces of perfect light absorption to improve color performances such as saturation and brightness. Thin-layer perfect absorbers with periodic hole arrays are designed at visible frequencies and the absorption peaks are tuned by simply adjusting the hole size and periodicity. Near perfect light absorption with high quality factors are obtained to realize high-resolution, angle-insensitive plasmonic color printing with high color saturation and brightness. Moreover, the fabricated metasurfaces can be protected with a protective coating for ambient use without degrading performances. The demonstrated structural color printing platform offers great potential for applications ranging from security marking to information storage.

Plasmonic metasurfaces^1^,^2^,^3^,^4^,^5^,^6^ have emerged as an exciting class of subwavelength architectures that exhibit extraordinary enhancement and confinement of optical fields with well-controlled intensity, phase and polarization of light beyond the diffraction limit^7^,^8^,^9^. By appropriately adjusting the structural geometries of metasurfaces, the plasmonic resonances of metasurfaces can be tuned from the visible regime through the infrared, serving as the foundation of many fascinating applications such as biochemical sensing^10^,^11^, photovoltaic solar cells^12^, optical imaging^13^, and color filtering^14^,^15^,^16^,^17^. Structural color printing based on plasmonic metasurfaces not only provides a much higher (about two-orders of magnitude) printing spatial resolution^18^ but also gives an attractive approach with reduced number of the needed materials^19^, compared to the traditional pigment-based color printing. Besides, structural color printing also has the advantages of lower cost, easier recycling, and higher reproduction fidelity and stability^20^.

Recently several strategies have been employed to realize structural colors, including interference effects induced by dielectric single layer^21^ or multilayers^22^, extraordinary optical transmission^23^ based plasmonic color filters^14^,^24^,^25^,^26^, and various kinds of plasmonic resonators above metallic reflectors^15^,^20^,^27^,^28^,^29^. Despite their potentials as next-generation color-printing technologies, however, the color printing performance of the previously reported approaches are limited by several factors, which will hinder their usage in practical applications. From the respect of materials used, for example, the interband transitions of gold limit the rendering of colors at wavelengths less than around 500 nm^20^,^30^, and the uncoated silver structures are susceptible to oxidation and sulphidation which may degrade the colors^29^. While aluminum nanostructures affords longer stability benefiting from impermeable natively coated oxide layer^18^,^19^,^30^,^31^,^32^,^33^, the higher loss of aluminum in the visible regime is a drawback for the color printing due to the fact that plasmonic resonances with broad bandwidth and low intensity contrast decrease severely the color brightness and saturation (or purity)^18^,^19^,^24^,^25^,^26^. From the respect of structures adopted, for example, reflection configurations based on localized plasmonic resonances exhibit weak angular dependence^19^ but suffer from the relatively large radiative damping due to low quality factor (*Q*-factor) dipolar resonances^18^,^19^,^27^. In general, in the existing approaches, plasmonic resonances with broad resonance peaks and low optical intensity contrasts significantly limit the performance of structural color printing.

For practical color printing applications where a high color brightness and saturation are preferred, it is necessary to achieve color filtering with low cross-talk^25^ and reasonably high optical reflection efficiency for three primary colors (cyan-magenta-yellow, CMY system). To meet this end, in this paper a simple but efficient structural color printing platform is introduced based on plasmonic metasurface with a thin-layer metal-dielectric-metal (MIM) structure, by which pure perfect absorption resonances with high *Q*-factor are tailored throughout the entire visible frequency range. Here silver is selected as the metallic material due to its low absorption loss in visible frequencies so that relatively sharper plasmonic resonances can be obtained for achieving better color saturation which is desirable in practical color printing applications. As a mechanical protection layer is always desired for use in exposed ambient color printing applications, the issues of oxidation and sulphidation of silver can be readily solved by coating the metasurface with a thin transparent polymer overlayer without significantly influencing the color performance. The resonance wavelength redshift induced by the polymer layer can be easily compensated by the geometrical adjustment.

## Results

### Plasmonic metasurfaces of perfect light absorption for color printing

**Figure 1:**
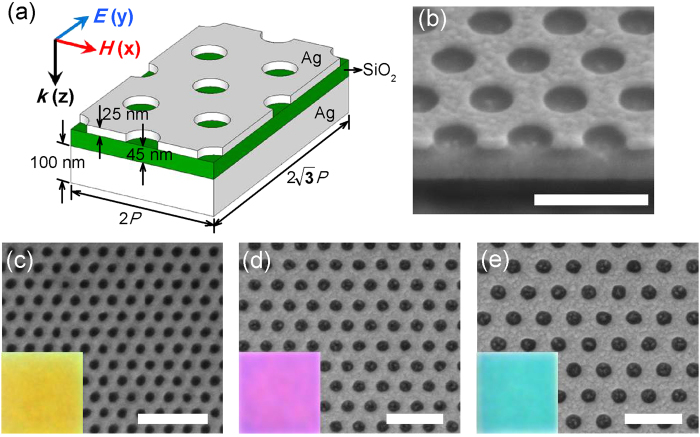
Plasmonic metasurfaces fabricated on silver-silica-silver three layer structures. (**a**) Schematic view of four unit cells for triangular-lattice circular hole arrays fabricated on the silver-silica-silver three layer structure. (**b**) An example of SEM cross-section image of the metasurface structure with period (*P*) of 320 nm and hole radius (*r*) of 100 nm. (**c**–**e**) SEM images of three metasurfaces with different lattice geometrical parameters (c: *P* = 130 nm, *r* = 35 nm; d: *P* = 200 nm, *r* = 50 nm; e: *P* = 260 nm, *r* = 65 nm). Insets: Optical reflection microscopy images of the entire 20 × 20 *μ*m^2^ circular hole arrays of triangular lattice. Scale bars: 500 nm.

Figure 1(a) illustrates the schematic diagram of the proposed plasmonic metasurfaces of perfect absorption consisting of three functional layers, including the top 25 nm silver thin film and the bottom 100 nm silver mirror, which are separated by a 45 nm silica dielectric spacer. Circular hole arrays of triangular lattice with period *P* and hole radius *r* are patterned on the top silver film. Figure 1(b) shows a cross-sectional scanning electron micrograph (SEM) image of a selected metasurface with *P* = 320 nm and *r* = 90 nm. The presence of the bottom silver mirror totally eliminates the transmittance (*T* = 0) of the metasurface due to the small penetration depth in silver (~20 nm at visible frequencies). The bottom 100 nm silver is deposited with the electron-beam evaporation system on top of a silicon wafer and subsequent layers are deposited with Kurt Lesker sputterer. A 5 nm silica overlayer is deposited finally to protect the triple-layer structure from degradation in air. The dependence of optical absorption resonance on the thickness of either the dielectric spacer or the top silver layer is investigated numerically as shown in Supplementary Figure S1. The top silver layer is patterned with circular hole arrays of triangular lattice by using the focused ion beam (FIB) milling (FEI Helios Nanolab 600 DualBeam system). Highly confined surface-plasmon polaritons (SPPs) will be excited by the periodic holes on the top silver thin film while the intermediate dielectric spacer will function as a bridge to couple the SPPs on two metal-dielectric interfaces^34^ to form an optical magnetic dipole resonance^11^,^35^. The coexistence of electric dipole resonance and magnetic dipole resonance in the plasmonic metasurfaces will result in matched impedance to the free space, leading to perfect light absorption and negligible reflection^36^. Additionally, it has been suggested that the hole array of a triangular lattice has a larger wavelength interval between the first two plasmonic resonances compared to a square lattice at the same lattice period^25^,^26^, which means the metasurface with triangular-lattice hole array will reduce the color cross-talk between different plasmonic resonances and therefore produce a purer color. Each fabricated hole array has a structural footprint of 20 × 20 *μ*m^2^ area which is sufficient for the optical reflection measurements. Representative SEM images of three metasurfaces with different geometrical parameters are shown in Fig. 1(c–e) and optical images obtained using bright-field microscope are shown in the insets. The SEM images reveal that the fabricated metasurfaces have well-defined circular holes with very low edge roughness. The optical images show a high degree of color saturation and homogeneity for bright colors of yellow, magenta and cyan, respectively.

**Figure 2:**
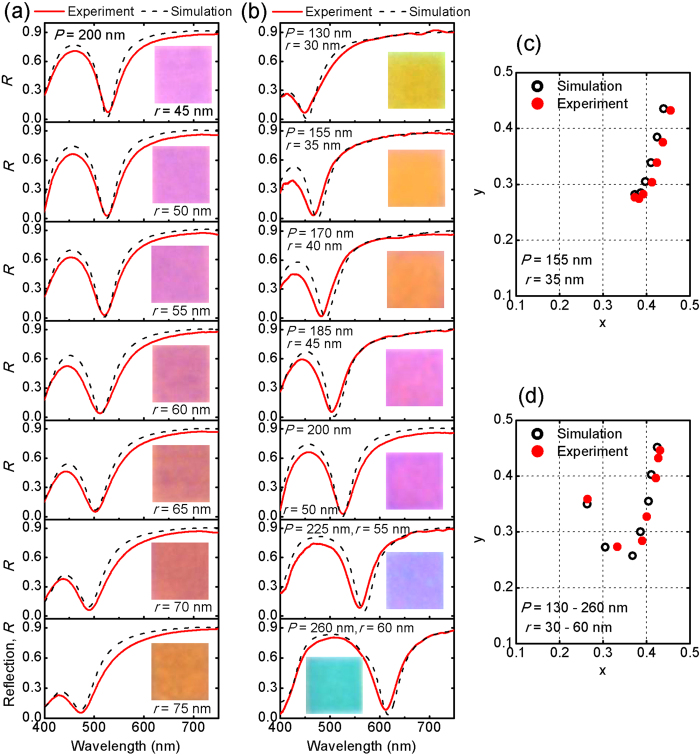
Analysis of reflection spectra and structural colors for the fabricated plasmonic metasurfaces. (**a**) Measured (red solid curves) and simulated (black dashed curves) optical reflection spectra of the metasurfaces with period *P* = 200 nm and hole radius *r* = 45 −75 nm (from top to bottom). Insets: The measured optical images of the fabricated 20 × 20 *μ*m^2^ metasurfaces. (**b**) Measured (red solid curves) and simulated (black dashed curves) optical reflection spectra of the metasurfaces with period *P* = 130 −260 nm and hole radius *r* = 30 −60 nm (from top to bottom). Insets: The measured optical images of the fabricated 20 × 20 *μ*m^2^ metasurfaces. (**c**) CIE 1931 chromaticity coordinates of measured (solid dots) and calculated (hollow dots) optical reflection spectra shown in panel (**a**). (**d**) CIE 1931 chromaticity coordinates for optical reflection spectra shown in panel (**b**).

The optical reflection spectra of the fabricated metasurfaces are characterized in the visible frequency range (400 −750 nm) with a home-built optical measurement system. At first the effects of the hole size on the optical absorption efficiency and color appearance are investigated. Seven metasurface structures consisting of triangular-lattice hole arrays with a constant period (*P* = 200 nm) but different hole radius (*r* = 45 −75 nm) are fabricated. Figure 2(a) gives the measured optical reflection spectra (red solid curves). As the hole radius increases from 45 to 75 nm in a 10-nm step, the absorption resonance wavelength is blue-shifted linearly from 527 to 473 nm and the full-width at half-maximum (FWHM) increases gradually, while the optical absorption at the resonance is always high. Especially, a narrow, near perfect absorption resonance (*A* ≈ 98%) is observed around the wavelength 520 nm for *r* = 50 −55 nm, which has a small FWHM of approximately 55 nm. Meanwhile, the corresponding optical image for each metasurface is shown as the inset along with the measured optical reflection curve. It is shown that the color changes gradually from bright magenta to orange as the hole radius increases from 45 to 75 nm. Note that such large absorption contrast within a narrow wavelength range leads to bright colors with high saturation. Numerical simulations (black solid curves) based on finite element method are used to identify the absorption resonance in the measured optical reflection spectra. Measured permittivities of silver and silica are employed in the simulations, resulting in a good agreement to the experimental results. The influence of different loss factors multiplied onto the imaginary part of the silver permittivity can be seen in Supplementary Figure S2.

In order to achieve a large tunable color range in the visible spectrum, the hole radius and lattice period are then varied simultaneously. Figure 2(b) shows the measured optical reflection spectra (red solid curves) with the period increases from 130 to 260 nm and the optimized hole radius from 35 to 65 nm. The unit cell with the period smaller than 300 nm is chosen here to reduce the influence of diffraction and to allow for wider viewing angles^20^. A rich color appearance is obtained as shown as the inset of each subfigure. Besides the bright magenta presented by the metasurface with 200 nm period shown in Fig. 2(a), another two primary colors of yellow and cyan as well as the intermediate colors between them are achieved by the metasurfaces with 130 nm period (top panel in Fig. 2(b)) and 260 nm period (bottom panel), respectively. All the simulated and measured optical reflection spectra have been converted to the CIE1931 chromaticity coordinates as shown in Fig. 2(c,d), illustrating the color gamut of the method. It is noticed that there are points all around the achromatic point, demonstrating the large degree of color range tuning ability^19^ and scaling property of the designed plasmonic metasurfaces.

### High-resolution plasmonic color printing with high brightness and saturation

**Figure 3:**
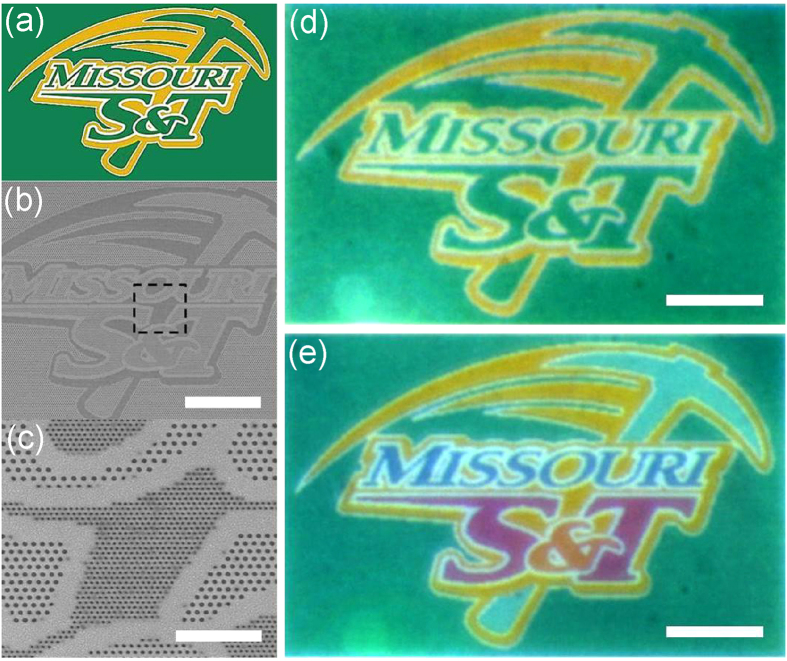
The demonstrated high-resolution plasmonic color printing with high brightness and saturation. (**a**) The original athletics mark image adapted with permission from The Curators of the University of Missouri. (**b**) SEM image of the fabricated pattern containing six different triangular lattices and corresponding colors shown in panel e. (**c**) SEM image of the area outlined in panel f. (**d**) Optical microscopy image of a plasmonic reproduction of the original mark image shown in panel e, containing only yellow and green colors. (**e**) Optical microscopy image of the plasmonic print presenting another four distinct colors (symbol ‘&’: orange, character ‘S, T’: magenta, pickaxe shape: cyan and word ‘MISSOURI’: navy blue) besides two original colors shown in panel d. Scale bars: 10 *μ*m (b, d and e); 2 *μ*m (c).

To demonstrate the suitability of the proposed metasurface platform and its visual performance in structural color printing related applications, the colored images of the athletics mark of Missouri University of Science and Technology are realized in Fig. 3. Both a miniature replication (51.6 × 33.3 *μ*m^2^, Fig. 3(d)) of the original pattern and a more colorful image (Fig. 3(e)) are prepared. By choosing the appropriate lattice period and hole radius in each pixel, the desired image colors (green and yellow) are reproduced with high fidelity and visual homogeneity. Moreover, another four distinct colors (symbol ‘&’: orange, character ‘S, T’: magenta, pickaxe shape: cyan and word ‘MISSOURI’: navy blue) displayed in Fig. 3(e) reveal high purity, brightness and large visual contrast in the produced colored image, benefiting from the strong absorption resonance supported in the fabricated metasurfaces. Furthermore, it is shown that bright colors can be produced by a tiny area containing only two pixels (as small as about 300 nm in width shown in Fig. 3(d,e)) and distinct colors are well preserved even at the sharp corners and boundaries of neighboring areas, exhibiting the ultrahigh color printing resolution and a high degree of reproducibility. This color demonstration proves that the proposed structural color printing based on metasurfaces of perfect absorption is capable of creating high-resolution pixels with sizes beyond the optical diffraction limit.

### Influence of protective polymer coating on the color printing

**Figure 4:**
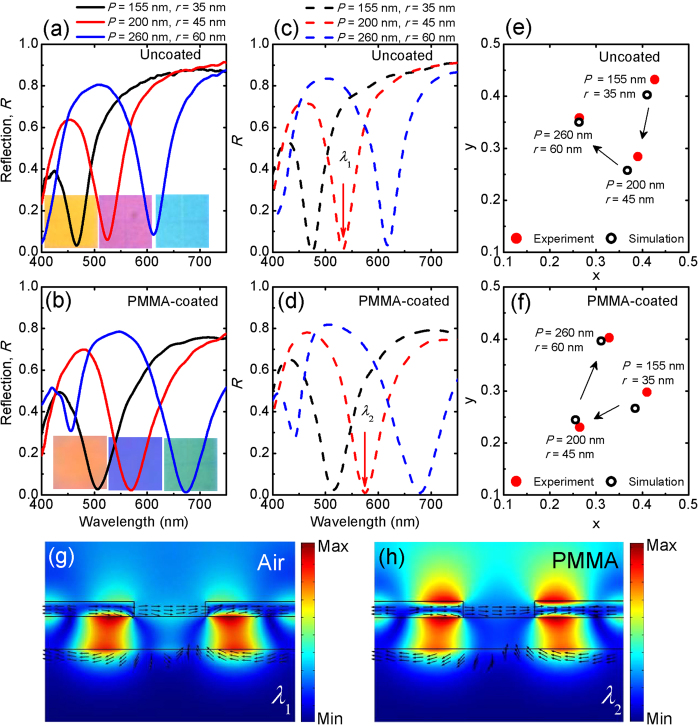
Comparison of reflection spectra and structural color for plasmonic metasurfaces without and with the protective polymer coating. Measured (**a**,**b**) and simulated (**c**,**d**) optical reflectance spectra of three selected metasurfaces without (**a**,**c**) and with (**b**,**d**) PMMA coating. (**e**) CIE 1931 chromaticity coordinates of uncoated metasurfaces. (**f**) Chromaticity coordinates of the three PMMA-coated metasurfaces. (**g**) Cross-section of the time-averaged magnetic field intensity (colored contours) and electric displacement (black arrows) distributions for the uncoated metasurface at the wavelength *λ*_1_ indicated in panel c. (**h**) Magnetic field intensity (colored contours) and electric displacement (black arrows) distributions for the PMMA-coated metasurface at the wavelength *λ*_2_ indicated in panel d.

In order to illustrate the applicability and flexibility of the plasmonic color printing platform for ambient use, the metasurfaces are further protected by spin-coating a 100 nm thick poly(methyl methacrylate) (PMMA) layer from chemical degradation (oxidization, sulphidation) and mechanical damages (scratching, fingerprints). Figure 4 shows a direct comparison between three selected uncoated metasurfaces and the PMMA-coated ones, including both the measured (Fig. 4(a,b), related bright-field microscope images shown in the insets of each figure) and simulated (Fig. 4(c,d)) optical reflection spectra. The additional PMMA protection layer does have limited influence on the observed colors, where an increase of the effective refractive index of the surrounding material leads to an observable redshift of the absorption resonance wavelength and a corresponding color change with extracted CIE 1931 chromaticity coordinates shown in Fig. 4(e,f). It should be noted that although the addition of protective coating induces some broadening of the absorption resonance bandwidth due to a weaker confinement of the optical field in the dielectric spacer^20^ as compared to the uncoated metasurface (Fig. 4(g,h)), the optical resonance properties including both the absorption magnitude and the *Q*-factor are almost unchanged. As an example for the metasurface with period 200 nm and radius 45 nm (red solid curves in Fig. 4(a,b)), the measured optical absorption at the resonance wavelength increases from 94% to 98% and the *Q*-factor (defined as *λ*_res_/FWHM) extracted from a Lorentz fitting decreases from 8.4 to 7.8, which are remarkable in plasmonic resonances^37^.

**Figure 5:**
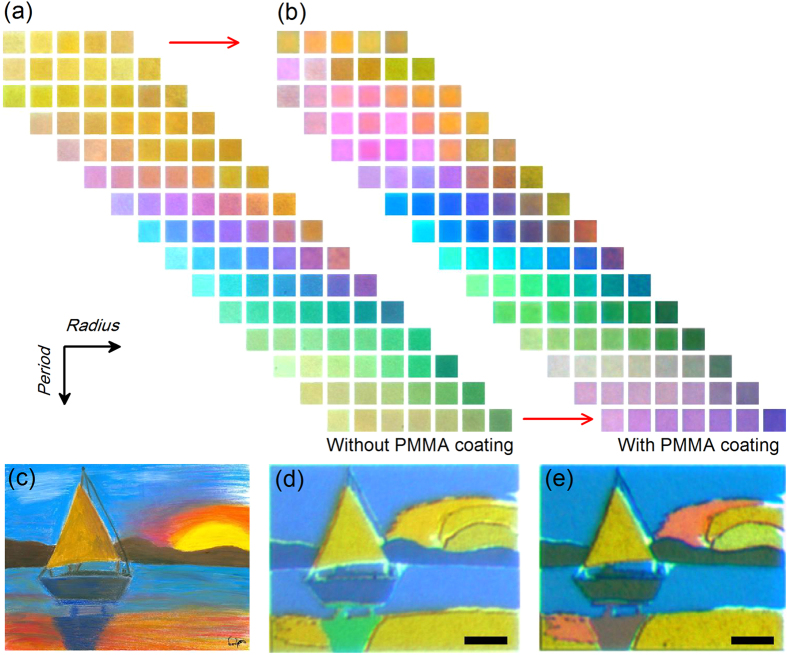
Color palette and reproduction of an artwork without and with the protective polymer coating. (**a**) The measured bright-field microscope images of uncoated metasurfaces with period varying from 120 to 320 nm and hole radius ranging from 25 to 115 nm. (**b**) The measured bright-field microscope images of the PMMA-coated metasurfaces. (**c**) Original image of a selected pastel painting obtained from a public domain resource. (**d**) Optical microscopy image of the uncoated plasmonic painting. Each colored area is visually uniform and exhibits bright color with high contrast, illustrating a high degree of accuracy in the fabrication process. (**e**) Optical microscopy image of the PMMA-coated plasmonic painting. The influence of spectral redshift induced by the PMMA coating is taken into account by choosing appropriate lattice period and hole radius, so that each color in the original painting is reproduced with high fidelity in the plasmonic painting. Scale bars: 10 *μ*m.

To further demonstrate the effect of PMMA coating on perceived color appearance, Fig. 5 shows the comparison of bright-field microscope images of color palettes for the uncoated metasurfaces and the PMMA-coated metasurfaces with hole radius between 25 −115 nm and period ranging from 120 to 360 nm, where more than 100 unique colors are included. It is shown that while the protective PMMA coating induces an influence on the perceived colors, there is no degradation of color brightness and saturation in the resultant color images. The gamut of the color palettes demonstrates that by appropriate design of hole size and lattice period, an ideal color production can be achieved with polymer protection^20^. Based on this idea, a structural color printed copy of a pastel painting from a public domain resource^38^ is fabricated in micrometer scale. For each color in the original painting shown in Fig. 5(c), an appropriate combination of lattice period and hole radius is selected to create the desired color by taking into account the influence of spectral redshift induced by the PMMA coating. The optical image of the PMMA-coated print reveals a high-quality color appearance (Fig. 5(e)) with a better color fidelity than the uncoated one (Fig. 5(d)). By taking the advantage of the high *Q*-factor of near perfect absorption of the metasurface structure, a relative broad color palette with high brightness and saturation is obtained by only changing the period and hole radius of the triangular lattices, without employing complicated color generation and mixing strategies such as in the case of aluminum color pixels^18^, where multiple nanostructures within a single pixel are included in order to compensate for the low *Q*-factor of the resonance. It is emphasized that the polymer-protected color printing method demonstrated here is well suited for practical applications, such as anti-counterfeit tag and security marking where color stability under mechanical or chemical influence is of paramount importance. As shown in Supplementary Figure S4, metasurfaces including three primary colors are exposed to ambient conditions for more than three months as a test of their durability and no signs of visible deterioration are observed.

### Angle-insensitive light absorption and color printing

The angular dependence of the optical reflection spectral response of the metasurface is investigated numerically for a selected structure with *P* = 160 nm and *r* = 45 nm for both *s*-polarized (electric field parallel to *y* axis) and *p*-polarized (magnetic field parallel to *y* axis) light (Supplementary Figure S3). The angle resolved chromaticity coordinates have also been calculated from the obtained optical reflectance spectra. It is shown that although a small absorption resonance splitting due to the lattice period is observed at incident angles larger than 30°, the maximum absorption remains higher than 95% even at large incident angles (70°) under both polarizations. Although the excitation of SPPs via grating coupling inherently constitutes a limitation on the angle independence of the resonance wavelength, the effect of lattice period on the spectral response is less pronounced for the designed metasurfaces as compared to the single layer transmission color filters^39^ due to the following reasons. According to the optical field distribution at the resonance wavelength shown in Fig. 4(g) (uncoated metasurface) and 4(h) (PMMA-coated metasurface), a strong magnetic dipole resonance resulting from anti-symmetric current flow is introduced^35^ in the metasurface structure which is responsible for the observed perfect light absorption when the thickness of dielectric spacer is small^40^. The magnetic dipole resonance is expected to remain robust at large incident angles for both *s*-polarized and *p*-polarized light for the adopted triangular lattices. This expectation is confirmed by the numerically obtained angle-resolved optical reflection spectra shown in Supplementary Figure S3 (a,b) which exhibit high absorption at wide angle range. As a result, the colors of selected reflection spectral curves (Supplementary Figure S3 (d,e), retrieved from angle resolved chromaticity coordinates using a MATLAB code) are demonstrated to be stable under large viewing angles up to 70°.

## Discussion

We have demonstrated the realization of high-performance structural color printing with high resolution, brightness and saturation by utilizing plasmonic metasurfaces of perfect light absorption. Due to the matched impedance to free space at the designated wavelength range, the metasurfaces present narrow-band and near unity absorption resonances in visible frequencies, yielding pure colors with high brightness and exhibiting in the meantime low dependences on polymer protection, incidence angles and polarizations. By simply scaling the period and hole radius of the triangular lattice in the metasurface design, wide color tunability across the entire visible frequency range has been achieved. We believe the demonstrated structural color printing platform based on the above plasmonic metasurfaces will open up the possibility of realizing high-performance, pigment-free color printing and relevant applications such as security marking and information storage.

## Methods

### Material deposition

The Ag-SiO_2_-Ag three-layer structure is deposited on top of a silicon wafer. The bottom 100 nm Ag layer is deposited using an electron-beam evaporator system. The SiO_2_ spacer and the top Ag layer are then deposited with sputtering method (Kurt J. Lesker), where Ag is deposited at the rate of 0.4 Å/sec and SiO_2_ is deposited at 0.2 Å/sec. The optical constant of both materials and the film thicknesses are characterized with the variable angle spectroscopic ellipsometry (VASE, J. A. Woollam Co. VB400/HS-190). The VASE measurements show that the optical constant of Ag matches the standard data of Johnson and Christy^41^ based on the fitting from a general oscillator model. The dielectric constant of SiO_2_ is fitted from the Cauchy dispersion relation.

### Device fabrication

The triangular lattices of circular holes are patterned on the top of the first metallic layer of the MIM structure via a one-step focused ion beam milling (FEI Helios Nanolab 600 DualBeam) with a gallium ion current of 9.7 pA and an accelerating voltage of 30 KeV. Then a thin layer of PMMA (950-A2 in anisole, Michrochem), a commonly used positive electron-beam resist, is spin-coated (2,000 rounds per min) on top of the fabricated nanostructures. We choose PMMA in the present work due to its chemical and mechanical stability and the accurate control of the thickness via control of the spin speed or molecule concentration used. The thickness of the coated PMMA polymer layer is also determined through X-ray reflectivity (Philips X’Pert-MRD) measurement.

### Optical characterization

We characterize the optical reflection spectra of all samples in the visible range (400 −750 nm) with a home-built optical measurement system. Halogen illumination is directed to the lattice area through a × 50 objective lens (numerical aperture of 0.42) and the reflected light from the metasurfaces is collected by the same objective lens and collimated through a pinhole defining the lattice area. Subsequently, the collected signal is directed to a fiber-coupled optical spectrometer (LR1, ASEQ instruments). The measured reflection spectra of all samples are then normalized by that of a silver coated mirror (THORLABS) which has optical reflection larger than 97.5% in the visible range.

### Numerical simulations

Finite element method (FEM) simulations are performed to obtain the optical reflection spectra and field distributions using the software (COMSOL Multiphysics). In the simulations, periodic boundary conditions are employed along the *x* and *y* axes to account for the periodic arrangement of the unit cells. Perfectly matched layers (PMLs) surrounded by scattering boundary condition faces are utilized along the propagation direction (perpendicular to the planar metasurfaces) to avoid multiple reflections due to geometry truncation. Measured values of the permittivity of Ag (imaginary part multiplied by factor of 2 to account for surface irregularities and additional losses induced by fabrication) and SiO_2_ are employed in the simulations. Besides, finite-difference time-domain (FDTD) simulations are also carried out to check the results obtained by FEM method and to calculate the angle resolved reflection spectra shown in Supplementary Figure S3.

## Additional Information

**How to cite this article**: Cheng, F. *et al.* Structural color printing based on plasmonic metasurfaces of perfect light absorption. *Sci. Rep.* **5**, 11045; doi: 10.1038/srep11045 (2015).

## Supplementary Material

Supplementary Information
