# Supplementary material for: Structural color printing based on plasmonic metasurfaces of perfect light absorption
Source: Sci Rep. 2015 Jun 5;5:11045. doi: 10.1038/srep11045 (PMC4457024; doi:10.1038/srep11045)
Supplement: Supplementary Information [file srep11045-s1.pdf]

Supplementary Information

Structural color printing based on plasmonic metasurfaces of perfect light absorption

Fei Cheng[1], Jie Gao[1,], Ting S. Luk[2], and Xiaodong Yang[1,*]*

[1]Department of Mechanical and Aerospace Engineering, Missouri University of Science and Technology, Rolla, MO 65409, USA

[2]Center for Integrated Nanotechnologies, Sandia National Laboratories, Albuquerque, NM 87185, USA

[*]Corresponding Email: yangxia@mst.edu and gaojie@mst.edu

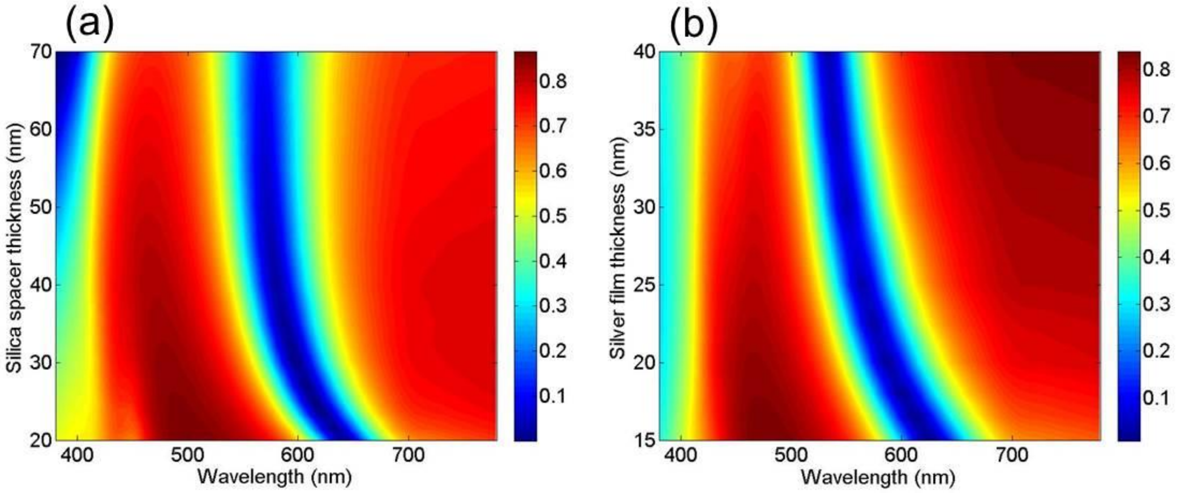

Figure S1 │ Simulated optical reflectance spectra of the PMMA coated samples for the case of period *P* = 200 nm, hole radius *r* = 50 nm. (a) Influence of dielectric spacer thickness (top silver thickness fixed at 23 nm) on the reflection spectra. (b) Influence of top silver thickness (dielectric spacer thickness fixed at 45 nm) on the reflection spectra. As the thickness of silica increases from 20 to 40 nm, the location of the reflection dip is blue-shifted about 60 nm (the resonance wavelength is from 650 to 590 nm) and then keeps almost at the same wavelength when the thickness of silica spacer further increases from 40 to 70 nm. Another blue-shift of the reflection dip is observed when the thickness of top silver layer increases from 10 to 40 nm. For both geometry variation circumstances, the resonant absorption remains at a high level (larger than 0.9) without obvious linewidth broadening, illustrating the robustness of the designed perfect absorbing metasurfaces.

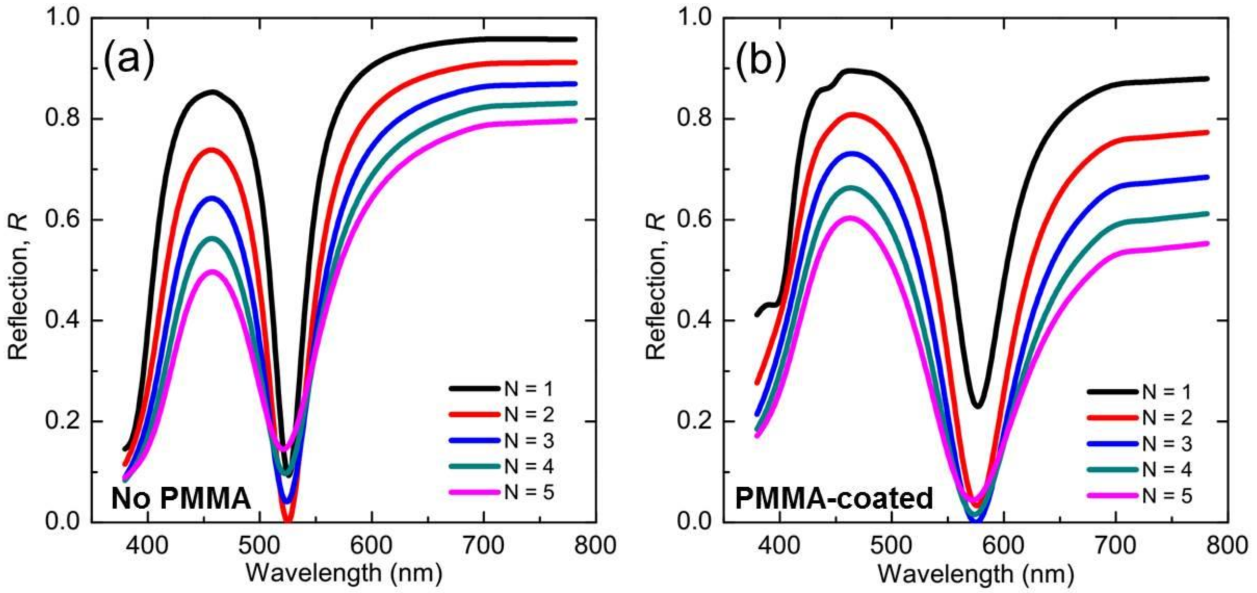

Figure S2 │ Simulated optical reflection spectra at normal incidence for the case of period *P* = 200 nm, hole radius *r* = 50 nm. (a) Reflection spectra of the uncoated metasurface with a factor *N* multiplied onto the imaginary part of the measured bulk silver permittivity. (b) Reflection spectra of the PMMA-coated (~100 nm) metasurface. Note that a strong resonance with nearly unity absorption is achieved when *N* is equal to two or three. This property is desirable in the realization of perfect absorbers and is also observed in other works[1]. In our simulations, we use a measured imaginary part multiplied by factor of $N = 2$ because it provides the best match to our experimental results.

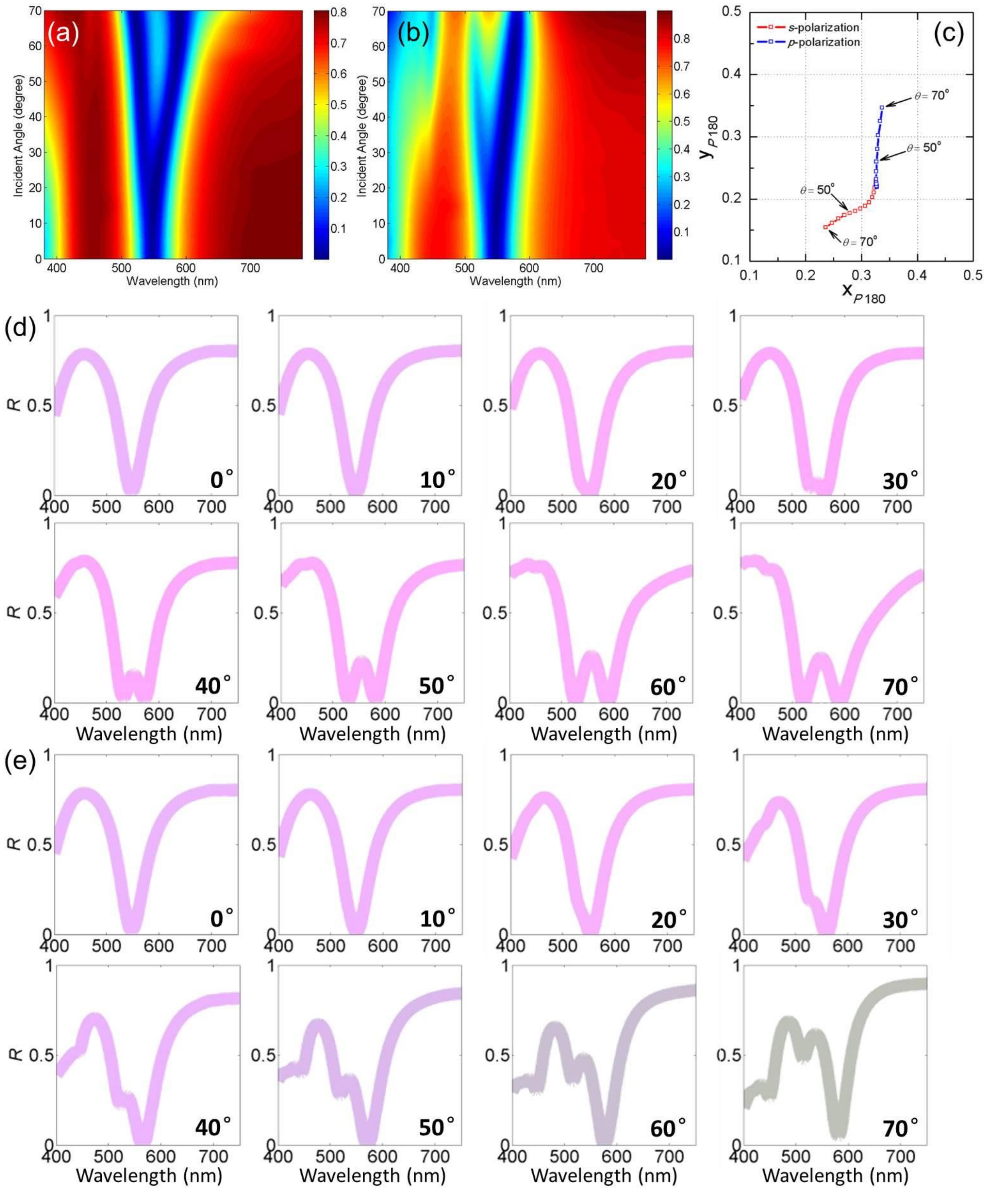

Figure S3│Angular dependence of reflection spectra for the case of period *P* = 180 nm, hole radius *r* = 45 nm. Simulated optical reflection spectra at oblique incident angles from 0° to 70° for both *s*-polarized (electric field parallel to *y* axis, panel a) and *p*-polarized (magnetic field

parallel to y axis, panel b) light for a PMMA-coated metasurface (period P = 180 nm, hole radius r = 45 nm). (c) Angular resolved CIE 1931 chromaticity coordinates for the metasurface for both s-polarized (red circles) and p-polarized (blue circles) light. (d) The optical reflection spectra from 0° to 70° for s-polarized light. The color of each reflection curve is retrieved from the standard CIE 1931 chromaticity space (using a MATLAB code) according to its chromaticity coordinate shown panel c. (e) Optical reflection spectra from 0° to 70° for p-polarized light with colors retrieved in the same way as in panel d. It is shown that the retrieved color is very stable for s-polarized light even at large incident angles up to 70° due to the near perfect absorption of the structure around the wavelength of 550 nm. For p-polarized incidence, the color is almost unchanged for incident angles below 40°, but the spectral variations lead to notable changes in color for incident angles larger than 50°.

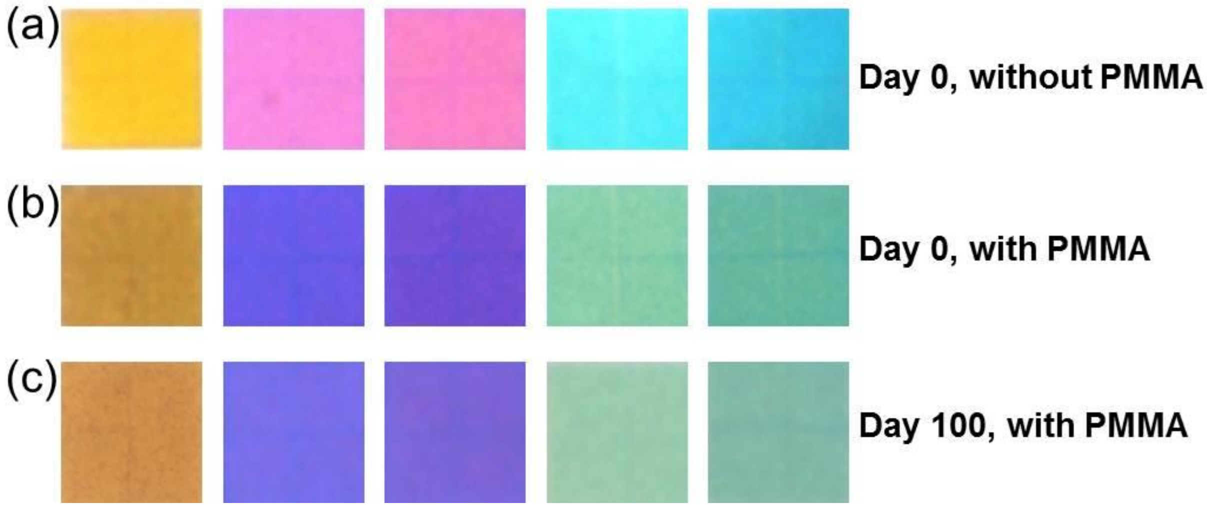

Figure S4 | Durability of PMMA-coated plasmonic colors. (a) Bright-field optical images of five selected structures (on the same wafer) upon fabrication without PMMA protection (Day 0). (b) Optical images of the structures upon fabrication with PMMA protection (Day 0). (c) Optical images of the structures with PMMA protection after 100 days. Due to oxidation and sulfidation of the perforated silver structure, the color performance exhibits visible signs of degradation within a week (another sample, data not shown). However, once the metasurfaces were protected by a thin layer of PMMA polymer, the structure remained remarkably stable without visible

color variation for a long time (> 3 months), showing a high degree of color retention and good durability.

1 Liu, N., Mesch, M., Weiss, T., Hentschel, M. & Giessen, H. Infrared Perfect Absorber and Its Application As Plasmonic Sensor. *Nano Letters* **10**, 2342-2348, doi:10.1021/nl9041033 (2010).
